# Rift Valley Fever Outbreak, Southern Mauritania, 2012

**DOI:** 10.3201/eid2002.131000

**Published:** 2014-02

**Authors:** Abdourahmane Sow, Ousmane Faye, Yamar Ba, Hampathé Ba, Diawo Diallo, Oumar Faye, Cheikh Loucoubar, Mohamed Boushab, Yahya Barry, Mawlouth Diallo, Amadou Alpha Sall

**Affiliations:** Unité des arbovirus et des Fièvres Hémorragiques Virales de l’Institut Pasteur de Dakar, Dakar, Senegal (A. Sow, Ousmane Faye, Oumar Faye, C. Loucoubar, A.A. Sall);; Unité d’Entomologie Médicale de l’Institut Pasteur de Dakar, Dakar (Y. Ba, D. Diallo, M. Diallo);; Institut National de Recherche et de la Santé Publique de Nouakchott, Nouakchott, Mauritania (H. Ba);; Ministère de la Santé de la République Islamique de Mauritanie, Mauritania (M. Boushab);; Centre National d’Etude et de Recherches Vétérinaires, Nouackchott (Y. Barry)

**Keywords:** Rift Valley fever, outbreak, re-emergence, field investigation, Mauritania, viruses, RVFV, Rift Valley fever virus, *Suggested citation for this article*: Sow A, Faye Om, Ba Y, Ba H, Diallo D, Faye Os, et al. Rift Valley fever outbreak, southern Mauritania, 2012. Emerg Infect Dis [Internet]. 2014 Feb [*date cited*]. http://dx.doi.org/10.3201/eid2002.131000

## Abstract

After a period of heavy rainfall, an outbreak of Rift Valley fever occurred in southern Mauritania during September–November 2012. A total of 41 human cases were confirmed, including 13 deaths, and 12 Rift Valley fever virus strains were isolated. Moudjeria and Temchecket Departments were the most affected areas.

## Abstract 2

Rift Valley Fever Outbreak, Mauritania, 2012

Rift Valley fever virus (RVFV; genus *Phlebovirus*, family *Bunyaviridae*) periodically causes outbreaks in humans and livestock (*1*), mostly in sub-Saharan Africa (*2*,*3*). Rift Valley fever (RVF) in humans is characterized by a mild, acute, febrile illness with spontaneous recovery, although 1%–2% of cases may evolve to more severe disease, such as acute hepatitis, encephalitis, retinitis, or a hemorrhagic syndrome (*4*,*5*). In Mauritania, RVF outbreaks have been reported repeatedly; the first occurred in 1987 after the building of the Diama dam, which had ecologic and environmental effects that favored a large-scale outbreak that resulted in 200 human deaths (*6*). Since then, RVF epizootics/epidemics have been reported in Mauritania in 1993, 1998, 2003, and 2010 (*7*–*10*). 

After 2-fold increase of rainfall in Mauritania during 2012 (annual total 269 mm compared with 137 mm in 2011, as measured at Temchekett station), abortions among pregnant domestic ruminant livestock were reported in the Tagant, Brakna, Trarza, Assaba, and Hodh-El-Gharbi regions of southern Mauritania, starting in September 2012 (Figure 1). By November 2012, many human patients with hemorrhagic fever, 13 of whom died, had been reported in the same regions. Preliminary biological investigation of suspected RVF cases confirmed 23 human cases; this finding led to a multidisciplinary field investigation in these regions during November 18–29, 2012. Here we report the results of this investigation and laboratory findings from this outbreak.

**Figure 1:**
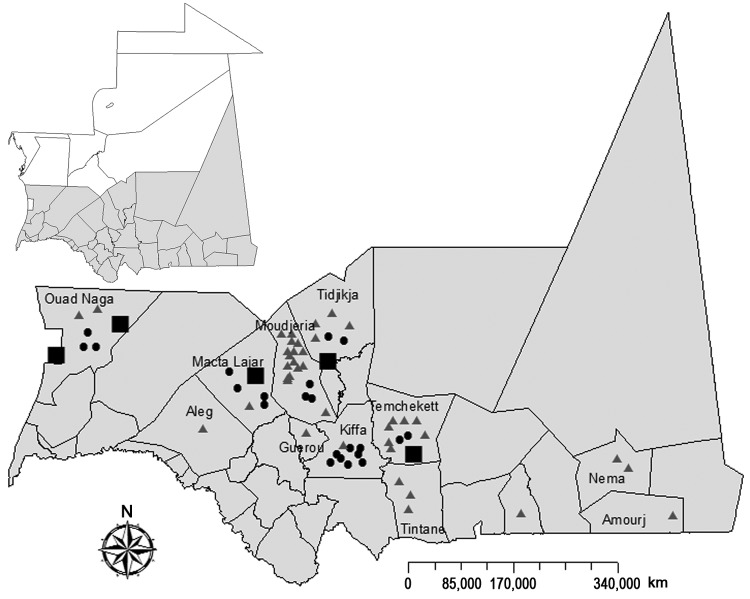
Geographic distribution of confirmed and probable cases of Rift Valley fever among humans and animals, southern Mauritania (gray shading), 2012. Triangles, confirmed human cases; dots, probable human cases; squares, confirmed animal cases.

## The Study

During the human outbreak investigation, the following case definitions were used: 1) a suspected case was illness in any patient living in Tagant, Brakna, Trarza, Assaba, and Hodh-El-Gharbi regions during September 1–November 29, 2012, that included fever and influenza-like syndrome, whether associated with bleeding or neurologic symptoms or not; 2) a probable case was a case in a patient with a suspected case who died before being tested for RVF infection markers; 3) a confirmed case was a case in a patient with a suspected case whose serum sample was positive for RVF IgM and/or who had positive results by RVF molecular assay. Contacts were defined as family members and neighbors of patients with confirmed and probable cases who showed risk for exposure to RVFV. 

For the entomologic investigation, arthropods were collected by using CDC light and animal-baited traps and aspirators, sorted by species and sex (for mosquitoes) or polyspecific pools (for the other arthropods) in the field on chill table, and stored in liquid nitrogen until testing for presence of virus. Human samples were tested for IgM and IgG and by reverse transcription PCR for RVFV (*11*). RVFV isolation from mosquitoes and human samples was attempted by inoculation in AP61 cell lines, followed by indirect immunofluorescence assay, as described (*12*). Partial sequencing of the G2 and nonstructural coding regions of the small segment of RVFV detected or isolated was also performed. Data were analyzed by using R software (www.r-project.org), χ^2^ test was used to compare difference between 2 proportions, and significance was set at p<0.05. Logistic regression was used to search for association between confirmed status and sociodemographic risk factors. Model accuracy was tested by using the Hosmer Lemeshow test; if p<0.05, the model was rejected, but otherwise, the model was considered adequate. 

**Figure 2:**
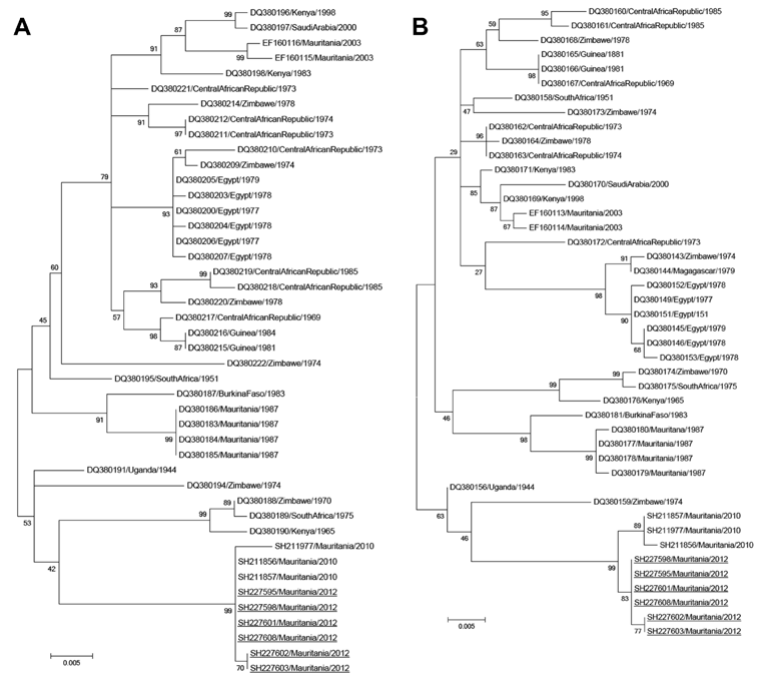
Phylogenetic trees for the medium (M) segments (680 nt) (A) and small (S) segments (531 nt) (B) of 6 Rift Valley fever virus isolates from southern Mauritania, 2012 (underlined), showing relationships among strains isolated from different localities and countries. The strains from 2012 grouped with strains isolated in northern Mauritania in 2010, which suggests re-emergence from an enzootic focus. GenBank accession numbers are KF648851–KF648856 for the M segments and KF648857–KF648862 for the S segments of the isolates identified in this study. GenBank accession numbers, countries, and year of isolation are given for the other strains. Scale bars indicate nucleotide substitutions per site.

A total of 288 persons had serum samples tested: 23 had confirmed cases, 47 had suspected cases, and 218 were contacts. Median age of those sampled was 24 (range 2–86) years. Forty-one (14%) persons had evidence of recent RVFV infection (20 positive for IgM, 15 by PCR, 6 both), and 24 (8%) had evidence of past infection (positive for IgG). Twelve RVFV strains were isolated from PCR-positive human samples, and phylogenetic analysis was performed on the medium and small genome segments for 6 of these isolates. These strains clustered in the West Africa lineage and formed a group with RVF strains that were isolated in northern Mauritania during the 2010 outbreak (*10*) (Figure 2).

The overall attack rate for this outbreak was 0.039% (Table 1), with 41 confirmed cases, including 13 deaths, and 22 probable cases. The outbreak may have started as early as July 2012, when 2 probable cases were reported at Ouad-Naga, but the first confirmed case was detected on September 9 (week 38) at the same location, after which confirmed and probable cases increased progressively from week 38 to week 41 (first week of October), when the peak of the epidemic was reached. Case reports then decreased progressively; the last confirmed and probable cases were reported during weeks 43 and 44, respectively (last 2 weeks of October). Letfatar and Marvek villages in Moudjeria and Temchekett Departments were the most affected areas during this outbreak. The attack rate was 26% (11 confirmed cases, including 2 deaths, and 2 probable cases) in Letfatar and 20% (5 confirmed cases and 1 probable case) in Marvek. In addition, 75% (18/24) of the patients who had positive results for RVF IgG (most [85.9%] adults) were found in Tagant region; IgG prevalence was 54% and 21% in Moudjeria and Tidjigja, respectively. 

**Table 1:** Attack rates and lethality of human Rift Valley fever cases, by region, sex, and age, southern Mauritania, November 2012

Category	No. confirmed cases (no. deaths)	No. probable cases	Attack rate, cases/ 1,000 population	Death rate, %	Total population
Region					
Moudjeria	16 (4)	3	0.39	25.0	48,751
Tiguidja	4 (3)	2	0.128	75.0	46,944
Tamcheket	7 (0)	2	0.22	0	40,887
Tintane	3 (1)	0	0.035	33.0	84,649
Kiffa	1 (0)	8	0.088	0	102,057
Macta lajar	2 (1)	4	0.095	50.0	62,857
Ouad Naga	2 (1)	3	0.143	50.0	34,898
Guerou	1 (1)	0	0.024	100.0	41,844
Aleg	1 (0)	0	0.011	0	88,077
Djigueni	1 (0)	0	0.017	0	58,619
Amourj	1 (1)	0	0.001	100.0	936,619
Nema	2 (1)	0	0.024	50.0	84,242
Sex					
M	18 (7)	10	0.036	38.9	782,614
F	23 (6)	12	0.041	26.1	847,830
Age group, y					
0–14	5 (0)	2	0.011	0	658,699
15–44	29 (12)	15	0.071	41.4	622,831
45–64	3 (0)	3	0.021	0	291,849
>65	4 (1)	2	0.105	25.0	57,065
Total	41 (13)	22	0.039	31.7	1,630,444

Persons 15–44 years of age were most affected during this RVF outbreak, representing 70.7% (29/41) of confirmed cases and 68.2% (15/22) of probable cases (Table 1). Housewives and shepherds had significantly higher attack rates than did persons in other occupational categories (36.6%; p = 0.09), probably because of exposure to potentially infected mosquitoes during agricultural work or direct contact with viremic livestock, infected tissues, and aborted animals. 

Analysis of signs and symptoms among patients with confirmed RFV showed that 73.2% (30/41) had mild signs and symptoms (fever, headache, arthralgia, and myalgia), whereas 26.8% (11/41) developed hemorrhagic symptoms, mainly epistaxis (6/11 [54.5%]) and hematemesis (2/11 [18.2%]). Patients with probable cases showed more severe signs of the disease, including gingival bleeding (43%), epistaxis (36%), gastrointestinal bleeding (21%), neurologic signs (14%), and petechiae (14%). Differential diagnosis also enabled the identification of 1 case of Crimean-Congo hemorrhagic fever in Moudjeria; 12 additional persons showed signs of past infection.

A total of 292 arthropods, including 152 mosquitoes belonging to 13 species, were collected during November at 12 sites (1 trap-night/site) (Table 2). *Aedes vexans*, *Culex poicilipes, Culex antennatus*, and *Mansonia uniformis* comprised 52.6% of the mosquitoes collected; these 4 species are known to be RVFV vectors in the subregion. No RVFV strains were isolated from mosquitoes, but the investigations were carried out after most breeding sites had dried up and after the start of a vector control campaign by the Mauritanian authorities, which limited the number of specimens collected. 

**Table 2:** Arthropods collected during investigation of Rift Valley fever outbreak, by region, southern Mauritania, 2012

Species	Region				Total
	Moudjeria	Ouad Naga	Tamcheket	Tidjikja	
Mosquitoes					
* Aedes sudanensis*	–	–	–	1	1
* Ae. vexans*	–	–	–	45	45
* Anopheles funestus*	–	–	–	1	1
* An. pharoensis*	7	–	–	–	7
* An. rhodesiensis*	–	–	8	–	8
*An. rhodesiensis* male	–	–	21	–	21
* An. rufipes*	15	–	–	–	15
* An. ziemanni*	5	–	–	–	5
* Culex antennatus*	6	–	–	13	19
* Cx. decens*	–	–	–	1	1
*Cx. decens* male	–	–	–	1	1
* Cx. neavei*	2	–	–	–	2
* Cx. poicilipes*	15	–	–	–	15
* Cx. quinquefasciatus*	–	3	–	1	4
*Cx. quinquefasciatus* male		6	–	–	6
* Mansonia uniformis*	1	–	–	–	1
Total no. mosquitoes	51	9	29	63	152
Other arthropods					
*Culicoides* spp.	6	–	1	12	19
*Phlebotomus* spp.	108	–	11	2	121
Total no. arthropods	165	9	41	77	292

*–, none found.

## Conclusions

An RVF outbreak in southern Mauritania during late 2012 consisted of 41 confirmed cases, including 13 deaths, and 22 probable cases. The extent of this outbreak is probably underestimated because some RVF cases may be asymptomatic or misdiagnosed as malaria (*13*); underdiagnosis could also explain why the case-fatality rate (31.7%) for this outbreak was higher than that for previous outbreaks in Mauritania in 1998 (16%) (*7*) and 2003 (6.67%) (*8*). Phylogenetic analysis suggests that this outbreak resulted from re-emergence from an enzootic focus; RVF isolates from 2012 are closely related to isolates obtained in 2010 in northern Mauritania (Figure 2), which suggests endemicity of RVF in Mauritania that can be maintained by vertical transmission (*14*).

To facilitate identification of RVFV among animals and humans in Mauritania, an active surveillance system should be implemented or reinforced in these areas, and RVF should be primarily suspected for nonmalarial acute febrile illness (*15*). In addition, the identification of 1 acute and 12 past Crimean-Congo hemorrhagic fever infections during the investigation indicates the co-circulation of the 2 viruses, as previously shown (*8*), and calls for systematic differential diagnostics or a syndromic approach to hemorrhagic fever surveillance to avoid a large-scale epidemic. 
